# srBERT: automatic article classification model for systematic review using BERT

**DOI:** 10.1186/s13643-021-01763-w

**Published:** 2021-10-30

**Authors:** Sungmin Aum, Seon Choe

**Affiliations:** 1 Institute of Science and Technology (KIST), 5, Hwarang-ro 14-gil, Seongbuk-gu, Seoul, Republic of Korea; 2 Division of Nano and Information Technology, University of Science and Technology (UST), Gajeong-ro, Yuseong-gu, 34113 Daejeon, Republic of Korea; 3 Konkuk University, 120, Neungdong-ro, Gwangjin-gu, Seoul, Republic of Korea; 4 Data Republic, 320-1, Gwangnaru-ro, Seongdong-gu, Seoul, Republic of Korea; 5 Division of Biomedical Informatics, Seoul National University College of Medicine, Seoul National University Biomedical Informatics (SNUBI), Seoul, 03080 South Korea

**Keywords:** Systematic review, Process automation, Deep learning, Text mining

## Abstract

**Background:**

Systematic reviews (SRs) are recognized as reliable evidence, which enables evidence-based medicine to be applied to clinical practice. However, owing to the significant efforts required for an SR, its creation is time-consuming, which often leads to out-of-date results. To support SR tasks, tools for automating these SR tasks have been considered; however, applying a general natural language processing model to domain-specific articles and insufficient text data for training poses challenges.

**Methods:**

The research objective is to automate the classification of included articles using the Bidirectional Encoder Representations from Transformers (BERT) algorithm. In particular, srBERT models based on the BERT algorithm are pre-trained using abstracts of articles from two types of datasets, and the resulting model is then fine-tuned using the article titles. The performances of our proposed models are compared with those of existing general machine-learning models.

**Results:**

Our results indicate that the proposed srBERT_my_ model, pre-trained with abstracts of articles and a generated vocabulary, achieved state-of-the-art performance in both classification and relation-extraction tasks; for the first task, it achieved an accuracy of 94.35% (89.38%), F1 score of 66.12 (78.64), and area under the receiver operating characteristic curve of 0.77 (0.9) on the original and (generated) datasets, respectively. In the second task, the model achieved an accuracy of 93.5% with a loss of 27%, thereby outperforming the other evaluated models, including the original BERT model.

**Conclusions:**

Our research shows the possibility of automatic article classification using machine-learning approaches to support SR tasks and its broad applicability. However, because the performance of our model depends on the size and class ratio of the training dataset, it is important to secure a dataset of sufficient quality, which may pose challenges.

**Supplementary Information:**

The online version contains supplementary material available at 10.1186/s13643-021-01763-w.

## Background

A systematic review (SR) is a literature review that involves evaluating the quality of previous research and reporting comprehensive results from all suitable works on a topic [1]. It is an efficient and reliable approach that enables the application of evidence-based medicine in clinical practice [2].

However, SRs involve robust analyses, which require significant time and effort; these requirements prevent the application of up-to-date results in clinical practice. As per the Cochrane Handbook for Systematic Reviews of Interventions [3], it is recommended that the last search of relevant research databases should be within 6 months before publication of an SR; however, on average, it takes 67.3 weeks from the registration of protocol to the publication of an SR [4].

Therefore, tools to automate parts of the SR process have been suggested based on the recent advances in natural language processing (NLP). Even though manual intervention is required wherever creativity and judgment are needed [2, 5, 6], technical tasks can be supported by automated systems, which result in higher accuracy, shorter research times, and lower costs [5–7]. Moreover, recent advanced machine-learning techniques in the field of NLP could lead to the development of new algorithms that can accurately mimic the human actions involved in each step of an SR.

Global evidence maps [8, 9] and scoping studies [10] are examples of techniques that were designed to support the logical construction of inclusion criteria for SRs. To remove duplicate citations, many citation managers use semi-automated deduplication programs [11, 12] and additional heuristic [13] or probabilistic string-matching algorithms. Nevertheless, such current support systems for SRs only tend to focus on comparatively simple and intuitive tasks.

In this study, we attempt to automate the screening task, which constitutes a significant portion of the entire SR process and requires a considerable amount of effort. Followed by data acquisition for an SR, the screening task is performed to retrieve all relevant literature based on a predefined research question [10]. Although most irrelevant documents are quickly screened based on their title and abstract, a significant number of documents still need to be reviewed. These error-prone and time-consuming tasks were expected to be avoided by means of recently proposed decision support systems [14, 15] which learn inclusion rules by observing a human screener [16, 17]. However, these systems were unable to achieve high precision scores and also involved many limitations. Despite the necessity of sufficient data for training, it is difficult to obtain a large amount of labeled data in a domain-specific field. Furthermore, it is difficult to apply domain-specific literature to existing NLP models, which are trained using general corpora, and various language data cannot be processed simultaneously using a single model. These limitations hinder the development of a practical screening model for an SR, where various sources in different languages should preferably be included in order to ensure a well-rounded analysis of all reported works.

To overcome these limitations, such as the shortage of training data composed of domain-specific multilingual corpora, we adopted the Bidirectional Encoder Representations from Transformers (BERT) [18] algorithm for the SR process and referred to it as srBERT.

By pre-training the model with abstracts of included articles that were extracted during data collection, the proposed method overcomes the deficiency of training data and yields improved performance, resulting in a higher efficiency than traditional SR workflows. In addition, it is a practical model suitable for SR analyses; it can simultaneously process heterogeneous data comprising various languages and is also applicable to other datasets for the creation of SRs.

## Methods

### Datasets

To train the proposed algorithm, we used two types of datasets comprising documents that had been collected during SRs performed in previous works [19–24]. DatasetA comprises 3268 articles retrieved for the theme of “moxibustion for improving cognitive impairment” [24, 25]. The first task using datasetA was to classify the included articles that satisfy the three theme criteria: (1) cognitive impairment as the target disease, (2) moxibustion therapy as the intervention, and (3) experimental design using animal models. The model learned whether the paper should be included in the SR based on its title, and the ground truth for this task was binary labels manually classified by our team.

However, the original datasetA posed a potential risk of distorting the performance of the algorithm due to an imbalanced class composition: from the 3268 articles, only 360 articles were included, which was a ratio of 9.08:1. To compensate for this issue and to address the problem of data reduction or duplication that could be caused by simple over-/undersampling, we created dummy data by replacing words in the excluded articles with essential keywords to satisfy the inclusion criteria. For example, if an excluded article verified the effect of “acupuncture” as an intervention approach, we created included article title by replacing “acupuncture” with “moxibustion.” In this manner, for the first dataset, we obtained a total of 1333 included articles, and the final ratio was 2.45:1.

The second dataset, datasetB, comprised 409 case studies that were aimed at verifying the efficacy of oriental medicine treatments for all diseases. The second task using datasetB was to extract the relations of elements (RE) from the title of the articles.

In particular, key elements in a title were classified according to their categories, after which the relationships between elements were defined. Because the articles included in datasetB were case studies on oriental medicine, the keywords were composed of diseases and treatments (acupuncture and herbal medicine). Subsequently, the relationship between elements was defined, such as companion therapy (for treatment-treatment) or target disease (for treatment-disease).

Although the first task could be applied directly to datasetA using its already created labels, it was practically difficult to reconstruct datasetA for use in the second task. Conversely, datasetB could not be used for the first task because it was a collection of case reports, thus not suitable for selecting one specific topic. Therefore, classification (task 1) and RE (task 2) could be applied to each dataset, independently.

### Model

srBERT, which is based on the BERT model [18], is a pre-trained language representation model for automatically screening included papers for an SR. As a contextualized word-representation model, such as ELMo [26] and CoVe [27], the BERT model is characterized by applying a masked language model and pre-training based on deep bidirectional representations obtained from unlabeled text [28].

Despite the advantages of the original BERT model [18], we considered the importance of applying domain-specific corpora and vocabulary for creating SRs. Furthermore, to minimize the overall effort of gathering additional training data, while maintaining the flow of the existing SR process, we decided to employ most of the data generated during SR creation.

**Fig. 1:**
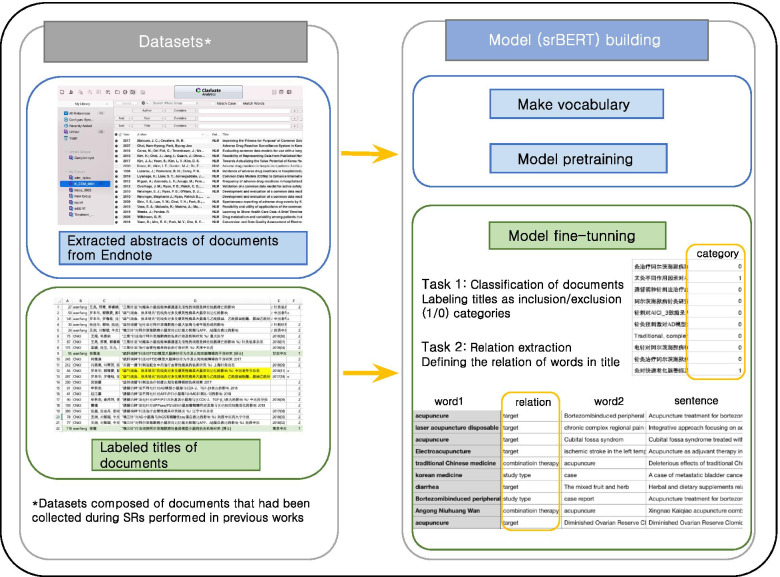
Procedure of building the srBERT model using datasets obtained via previous SRs. The abstracts of documents downloaded in Endnote are used to create the model vocabulary and pre-train the model. Data categorized as “Title,” which were obtained through manual screening, were used for the fine-tuning of srBERT. SR, systematic review; BERT, Bidirectional Encoder Representations from Transformers

**Fig. 2:**
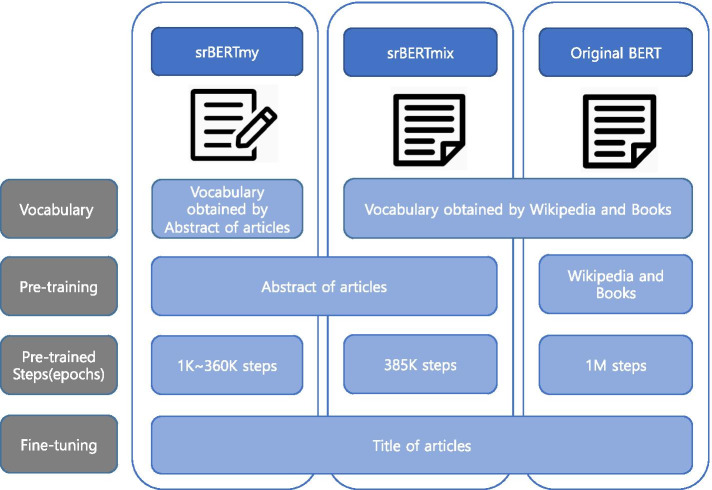
Compositions of the three BERT models. srBERT was pre-trained with domain-specific literature data, whereas the original BERT model was pre-trained using Wikipedia and books. srBERT_my_ used the vocabulary created by domain-specific literature data, whereas srBERT_mix_ used that of the original BERT model. All three models were fine-tuned using titles from literature data.. SR, systematic review; BERT, Bidirectional Encoder Representations from Transformers

Therefore, we pre-trained and fine-tuned srBERT using domain-specific documents that had previously been collected as corpus. The process of building the model using the dataset is illustrated in Fig. 1. Depending on the data used for pre-training, the models could be divided into srBERT_my_, srBERT_mix_, and original BERT. srBERT_my_ was pre-trained using abstracts of included articles with a vocabulary obtained via WordPiece tokenization [29] of the articles, whereas srBERT_mix_ was pre-trained using the same dataset as srBERT_my_, but it used the same vocabulary as the original BERT model. Figure 2 highlights the differences in composition of the three BERT models. After pre-training, the three models were fine-tuned using the titles of included articles.

### Fine-tuning the srBERT model

To enhance the applicability of a pre-trained srBERT model for given data and to verify its classification performance, all three models were fine-tuned and evaluated through classification tasks or extraction of element relationships from the titles of included articles.

## Results

### Model

#### Base model

In this study, we used the BERT-Base Un-normalized Multilingual Cased model, which was released on November 23, 2018; this model comprised 12 layers, 768 hidden, 12 heads, and 110 M parameters, covering 104 languages. Additional file 1 shows the hyperparameter values optimized for the model in more detail.

#### Fine-tuning model hyperparameters

The proposed srBERT was pre-trained using the Google Cloud Platform, which is typically used for large-scale experiments that need to be run on Tensor Processing Units (TPUs). We used eight NVIDIA V100 (32 GB) TPUs for pre-training our model. Approximately 5 days was required to pre-train each srBERT model. Furthermore, because the fine-tuning process was more computationally efficient than pre-training the model, we used a Google collaboration service to fine-tune srBERT for each classification task described earlier. For this fine-tuning, we tested the performance of the model with various combinations of hyperparameters to determine the one with the highest performance. Model performance was tested using max_seq_length of 128 and 256; training batch sizes of 4, 8, 32, 64, and 128; and learning rates of 1 × 10^−4^, 2 × 10^−6^, and 3 × 10^−5^.

#### Experimental setup

As previously specified, the original BERT model, which forms the basis of the proposed model, is pre-trained using English language articles from Wikipedia and Books Corpora for 1 M steps. The srBERT_my_ model was pre-trained using each dataset from steps 1 K to 400 K as learning epochs; 250 K and 355 K pre-training steps were found to be optimal for the first task, whereas 100 K steps were found to be optimal for the second task. Fine-tuning the proposed srBERT model for both tasks required less than an hour because the size of the training data is significantly smaller than the size of the data used for pre-training.

#### Experimental results

We tested our model on two types of tasks and compared the performances to those of existing models. Task 1 included article classification performed in both the original datasetA and the adjusted datasetA. Task 2 consisted of extracting relationships from the original datasetB. On average, the proposed srBERT models achieved better performance than the state-of-the-art models for all evaluated tasks; in particular, the srBERT_my_ model achieved the highest performance in terms of almost every performance index, including accuracy, precision, recall, F1 score, and area under the receiver operating characteristic curve (AUC).

For the first task in the original datasetA, the srBERT_my_ model, pre-trained using 250 K steps, exhibited the best performance, with an accuracy of 94.35%, F1 score of 66.12, and AUC of 0.77. Among existing models, the K-neighbors model exhibited the highest accuracy of 90.1% (Table 1). However, for the original datasetA, despite high accuracies of up to 90%, none of the models achieved an AUC exceeding 60, except for the srBERT_my_ model. This was attributed to data imbalance. In contrast, improvements in precision and recall scores, accompanied by a decrease in accuracy, were observed for every model when using the adjusted datasetA. In particular, the srBERT_my_ model trained on 355 K steps outperformed all other models, with an accuracy of 89.38%, AUC of 0.9, and F1 score of 78.46. This was followed by the original BERT model, which exhibited a performance similar to that of srBERT_my_. Table 2 lists the model performances for the title screening task.

**Table 1:** Performance of the models for the first task of article screening using the original datasetA

	srBERT_my250K_	srBERT_mix_	Original BERT	K-neighbors	SVC	DecisionTree	RandomForest	Adaboost	MultinomialNB
AUC	76.785	50.000	50.685	57.985	50.000	57.449	53.650	55.097	50.000
Accuracy	94.353	89.945	90.083	90.083	89.945	89.118	89.945	90.358	89.945
Precision	83.333	0.000	100.000	52.000	0.000	40.620	50.000	61.538	0.000
Recall	54.795	0.000	13.60	17.808	0.000	17.808	8.219	10.959	0.000
F1	66.116	0.000	26.84	26.531	0.000	24.762	14.118	18.605	0.000

*SR* systematic review, *BERT* bidirectional encoder representations from transformers, *srBERT*_*my250K*_ srBERT_my_ model trained for 250 K steps, *AUC* area under the curve, *SVC* support vector classification, *MultinomialNB* multinomial naive Bayes model

**Table 2:** Performance of the models for the first task of article screening using the adjusted datasetA

	srBERT_my355K_	srBERT_mix_	Original BERT	K-neighbors	SVC	DecisionTree	RandomForest	Adaboost	MultinomialNB
AUC	90.016	50.000	50.000	58.976	50.000	66.258	66.431	57.319	53.158
Accuracy	89.380	77.120	71.009	75.590	77.123	77.594	78.420	78.066	77.241
Precision	68.900	0.000	0.000	44.715	0.000	51.163	53.416	56.061	51.515
Recall	91.100	0.000	0.000	28.351	0.000	45.361	44.330	19.072	8.763
F1	78.460	0.000	0.000	34.700	0.000	48.087	48.451	28.462	14.978

*SR* systematic review, *BERT* bidirectional encoder representations from transformers, *srBERT*_*my355K*_ srBERT_my_ model trained for 355 K steps, *AUC* area under the curve, *SVC* support vector classification, *MultinomialNB* multinomial naive Bayes model

For the second task, which involved extracting relationships between the words in article titles, the srBERT_my_ model, which was trained on 100 K steps, showed better performance than the other sub-models, achieving an accuracy of 93.5% with a loss of 27%; this is similar to the performance of the original BERT model, which achieved an accuracy of 92% with a loss of 23% (Table 3).

**Table 3:** Performance of srBERT_my_ with respect to the learning steps for the second task (relation extraction) using datasetB

	srBERT_my50K_	srBERT_my100K_	srBERT_my150K_	srBERT_my200K_	srBERT_my250K_	srBERT_my300K_	srBERT_my350K_	srBERT_mix_	Original BERT
Accuracy	0.922	0.935	0.896	0.909	0.922	0.909	0.909	0.922	0.922
Loss	0.337	0.270	0.542	0.540	0.328	0.658	0.658	0.309	0.232

*SR* systematic review, *BERT* bidirectional encoder representations from transformers, *srBERT*_*my#K*_ srBERT_my_ model trained for # K steps

## Discussion

Even though SR is a comprehensive and reliable approach for clinical research, due to the time consumption required for the reviewing process, most SRs are already outdated by the time of publication [2], and the recommended update interval is difficult to satisfy [30]. Among the tasks where automation tools could be supported for SR creation, we focused on the appraisal stage for automatic sorting of trials into predefined categories of interest.

Our challenge was to manage insufficient training data in the form of multilingual documents. Therefore, we devised a multilingual BERT-based model, which is pre-trained and fine-tuned using documents obtained during the SR process. With only minimal architectural modifications, the srBERT model can be used in various downstream text-mining tasks. For both screening and RE, the proposed srBERT_my_ model achieved superior performance compared with other models, followed by the original BERT model.

Because the screening task only filtered out sparse data from a large amount of exclusion data [10], data imbalance was another challenge. Thus, we adjusted the class ratio of datasetA by generating dummy data; the model fine-tuned using the new data showed improved performance in terms of precision, recall, F1 score, and AUC metrics. For both evaluation datasets, the proposed srBERT_my_ model, trained on abstracts and new vocabulary data, outperformed all other models in terms of all performance scores. However, the original BERT and srBERT_mix_ models, pre-trained on abstracts with provided vocabulary, exhibited a higher risk of not being trained properly, with an AUC of 0.5 and with precision and recall values of 0. In the second task, the srBERT models achieved better performance than the original BERT model, with an accuracy of more than 90%, which demonstrated the effectiveness of the srBERT models for RE.

To attain optimal performance, we compared the changes in the performance of the models for different learning epochs. For example, for the bioBERT model [31], which had been trained using biomedical corpus, it was reported that 200 K and 270 K pre-training steps were optimal. For our proposed srBERT models, the performance difference depended on the task and applied dataset; for the first task with the original and generated datasets, the srBERT_my_ models trained with 250 K and 355 K steps, respectively, exhibited superior performance, while for the second task, the srBERT_my_ model trained with 100 K steps, was found to be optimal. Nevertheless, the models pre-trained with more than 50 K steps showed similar stability and excellent performance.

Through our work, we determined the efficiency and feasibility of the proposed srBERT model in supporting SR creation. Aside from its state-of-the-art performance compared with other models, the srBERT model also had the potential to be used for various SR tasks. For SRs that have already been performed, the proposed model could be used to screen newly updated data. It can also be applied for creating new SRs even for different subjects, as long as a similar corpus is used.

However, there were limitations to consider in our model. We designed a multilingual model, in accordance with the aim of SR, analyzing as many varied articles as possible without language restriction, while also pursuing the efficiency of model by processing them at once. In testing two datasets, our model worked well on both; datasetA consisted of both Chinese and English articles (Chinese accounted for more than 90% of the data), and datasetB was composed of only English articles. Considering the English terminology used in non-English papers, the universality of our model was meaningful.

Nevertheless, the model trained on multilingual data implied potential biases reducing the confidence of performance. It was difficult to assess whether the model had been trained according to each language’s characteristics or which language was better optimized for it. Our model showed different levels of training and performance depending on the language. The first model, which had been trained with a high proportion of Chinese-oriented data, tended to have a poor accuracy of classification of English data.

Despite the efficiency of the multilingual model, improvement of performance in accuracy and reliability could be obtained by the model optimized in each language; more sophisticated models to compensate for this point are expected.

In addition, model vulnerabilities whose precision is biased by the observed data could be raised due to the limited training datasets. Based on the prediction results obtained using the different models, we observed the learning performance to be poor in the following common cases: (1) data included new words and abbreviations that were not part of the training vocabulary; (2) cases with ambiguous titles, wherein the content of the abstracts or the full texts of the articles were required; (3) multilingual papers, such as those that include both English and Chinese; (4) cases where data were labeled incorrectly during data processing and which were then included in the dataset.

Excluding the technical issue such as ambiguity of the title and labeling errors, the learning performance was significantly influenced by the sufficiency of the training datasets that secured various terminology. It is an inevitable challenge of NLP model in specialized domain, even though we tried to overcome it while it still remained as a limitation. Along with the increasing demand for NLP in various domains, model optimization could be improved by cooperation of experts to build their own corpus for their field. For example, there are BERT models that have been trained only with corpora from the medical field, such as bioBERT [31] and clinical BERT [32]. If each researcher pre-trained their own BERT model appropriately to their field of interest, they could reuse it by additionally training only detailed topics. We expect srBERT can participate in and contribute to the work.

Meanwhile, there are concerns regarding the usability of models for general SR tasks due to their dependency on the pre-training data. Although the subject of SR is distinct from previous studies, the model pre-trained with a wide range of resources that share keywords in a common domain can be widely reused, optimizing the individual SR only by changing the last fine-tuning step. Since the fine-tuning is inexpensive in terms of computational cost compared to the pre-training process, this form of transfer learning allowed researchers to take advantage of the powerful deep neural network models without having access to a high-end computing environment.

Although we did not experience such problem, but it may be possible that direct fine-tuning of pre-trained model may not always amount to an excellent performance. Some data might be detrimental to the performance increase; therefore, there applying a systematic means of data valuation [33, 34] to filter out certain data may be beneficial. This could potentially allow more efficient transfer learning, which in turn increase the usability of the models in tasks 1 and 2 for general SR tasks. We consider this to be one of the most promising paths to explore in future.

## Conclusions

In this study, we proposed the srBERT model for the classification of articles to support the SR process. The superior performance achieved by the srBERT model demonstrated its efficacy for data screening; in addition, the importance of pre-training using domain-specific corpora for article classification was also highlighted. Although it required minimal task-specific architectural modification, the proposed srBERT model outperformed existing models in text mining for SR tasks, such as data classification and RE.

Our research demonstrated the possibility of automatically classifying articles to support SR tasks, and the broad applicability of BERT-based models with reusable structures and processes. However, because the performance of our proposed model depended on the size and class ratio of the dataset used, it was important to secure a high-quality training dataset to ensure satisfactory classification performance.

## Supplementary Information

Additional file 1

## Data Availability

The pre-trained data format and weights of srBERT are available at https://github.com/SEONCHOE/.

## Abbreviations

SR: Systematic review
NLP: Natural language processing
BERT: Bidirectional Encoder Representations from Transformers
TPU: Tensor Processing Unit
AUC: Area under the curve
RE: Relation extraction
SVC: Support vector classification

## References

[1] Clarke M, Hopewell S, Chalmers I (2007). Reports of clinical trials should begin and end with up-to-date systematic reviews of other relevant evidence: a status report. J R Soc Med.

[2] Cohen A, Adams C, Yu C, Yu P, Meng W, Duggan L, et al. Evidence-based medicine, the essential role of systematic reviews, and the need for automated text mining tools. In Proceedings of the 1st ACM International Health Informatics Symposium, 2010; doi: 10.1145/1882992.1883046

[3] Higgins JPT, Thomas J, Chandler J, Cumpston M, Li T, Page MJ, Welch VA (editors). Cochrane Handbook for Systematic Reviews of Interventions version 6. Cochrane, 2019. Available from www.training.cochrane.org/handbook. 10.1002/14651858.ED000142 PMC10284251 31643080

[4] Borah R, Brown AW, Capers PL, Kaiser KA (2017). Analysis of the time and workers needed to conduct systematic reviews of medical interventions using data from the PROSPERO registry. BMJ Open.

[5] Tsafnat G, Dunn A, Glasziou P, Coiera E (2013). The automation of systematic reviews. BMJ.

[6] Wallace BC, Dahabreh IJ, Schmid CH, Lau J, Trikalinos TA (2013). Modernizing the systematic review process to inform comparative effectiveness: tools and methods. J Comp Eff Res.

[7] O'Connor AM, Tsafnat G, Gilbert SB, Thayer KA, Wolfe MS (2018). Moving toward the automation of the systematic review process: a summary of discussions at the second meeting of International Collaboration for the Automation of Systematic Reviews (ICASR). Syst Rev.

[8] Bragge P, Clavisi O, Turner T, Tavender E, Collie A, Gruen R (2011). The global evidence mapping initiative: scoping research in broad topic areas. BMC Med Res Methodol.

[9] Snilstveit B, Vojtkova M, Bhavsar A, Stevenson J, Gaarder M (2016). Evidence & gap maps: a tool for promoting evidence informed policy and strategic research agendas. J Clin Epidemiol.

[10] Arksey H, O’Malley L (2005). Scoping studies: towards a methodological framework. Int J Soc Res Methodol.

[11] Qi X-S, Bai M, Yang Z-P, Ren W-R (2013). Duplicates in systematic reviews: a critical, but often neglected issue. World J Meta Anal.

[12] Qi X, Yang M, Ren W, Jia J, Wang J, Han G, Fan D (2013). Find duplicates among the PubMed, EMBASE, and cochrane library databases in systematic review. PLOS One.

[13] Jiang Y, Lin C, Meng W, Yu C, Cohen AM, Smalheiser NR (2014). Rule-based deduplication of article records from bibliographic databases. Database.

[14] Kiritchenko S, de Bruijn B, Carini S, Martin J, Sim I (2010). ExaCT: automatic extraction of clinical trial characteristics from journal publications. BMC Med Inform Decis Mak.

[15] Thomas J, McNaught J, Ananiadou S (2011). Applications of text mining within systematic reviews. Res Synth Method.

[16] Ananiadou S, Rea B, Okazaki N, Procter R, Thomas J (2009). Supporting systematic reviews using text mining. Soc Sci Comput Rev.

[17] Wallace BC, Small K, Brodley CE, Lau J, Trikalinos TA (2012). Deploying an interactive machine learning system in an evidence-based practice center: abstrackr. Proceedings of the 2nd ACM SIGHIT International Health Informatics Symposium.

[18] Devlin J, Chang M-W, Lee K, Toutanova K. BERT: pre-training of deep bidirectional transformers for language understanding. Preprint at https://arxiv.org/pdf/1810.04805.pdf (2019).

[19] Wang P, Yang J, Liu G, Chen H, Yang F (2010). Effects of moxibustion at head-points on levels of somatostatin and arginine vasopressin from cerebrospinal fluid in patients with vascular dementia: a randomized controlled trial. Zhong Xi Yi Jie He Xue Bao.

[20] Chen H, Wang P, Yang J, Liu G (2011). Impacts of moxibustion on vascular dementia and neuropeptide substance content in cerebral spinal fluid. Zhongguo Zhen Jiu.

[21] Li Y, Jiang G. Effects of combination of acupuncture and moxibustion with Chinese drugs on lipid peroxide and antioxidase in patients of vascular dementia. World J Acupunct Moxibustion. 1998;1.

[22] Liang Y. Effect of acupuncture-moxibustion plus Chinese medicinal herbs on plasma TXB2, 6-Keto-PGF1α in patients with vascular dementia. World J Acupunct Moxibustion. 1999;4;245–8.

[23] Wang Pin YJ, Yang F, Chen H, Huang X, Li F. [Clinic research of treating vascular dementia by moxibustion at head points]. China J Traditional Chin Med Pharm. 2009,24(10):1348–50.

[24] Choe S, Cai M, Jerng UM, Lee JH (2018). The efficacy and underlying mechanism of moxibustion in preventing cognitive impairment: a systematic review of animal studies. Exp Neurobiol.

[25] Aum S, Choe S, Cai M, Jerng UM, Lee JH (2021). Moxibustion for cognitive impairment: a systematic review and meta-analysis of animal studies. Integr Med Res.

[26] Peters ME, Neumann M, Iyyer M, Gardner M, Clark C, Lee K, Zettlemoyer L. Deep contextualized word representations. Preprint at https://arxiv.org/pdf/1802.05365.pdf (2018).

[27] McCann B, Bradbury J, Xiong C, Socher R. Learned in translation: contextualized word vectors. Preprint at https://arxiv.org/pdf/1708.00107.pdf (2018).

[28] Vaswani A, Shazeer N, Parmar N, Uszkoreit J, Jones L, Gomez AN, et al. Attention is all you need. Preprint at https://arxiv.org/pdf/1706.03762.pdf (2017).

[29] Wu Y, Schuster M, Chen Z, Le QV, Norouzi M, Macherey W, et al. Google’s neural machine translation system: bridging the gap between human and machine translation. Preprint at https://arxiv.org/pdf/1609.08144.pdf (2016).

[30] Jaidee W, Moher D, Laopaiboon M (2010). Time to update and quantitative changes in the results of Cochrane pregnancy and childbirth reviews. PLoS One.

[31] Lee J, Yoon W, Kim S, Kim D, Kim S, So CH, Kang J (2020). BioBERT: a pre-trained biomedical language representation model for biomedical text mining. Bioinform.

[32] Alsentzer E, Murphy JR, Boag W, Weng W-H, Jin D, Naumann T, McDermott MBA. Publicly available clinical BERT embeddings. Preprint at https://arxiv.org/abs/1904.03323.pdf (2019).

[33] Ghorbani A, Zou J: Data Shapley: equitable valuation of data for machine learning. Preprint at https://arxiv.org/abs/1904.02868.pdf (2019).

[34] Aum S. Automatic inspection system for label type data based on Artificial Intelligence Learning, and method thereof. Korean Intellectual Property Office, Registration Number : 1021079110000 (2020).

