# Supplementary material for: srBERT: automatic article classification model for systematic review using BERT
Source: Syst Rev. 2021 Oct 30;10:285. doi: 10.1186/s13643-021-01763-w (PMC8556883; doi:10.1186/s13643-021-01763-w)
Supplement: Supplementary file 1 — Additional file 1 [file 13643_2021_1763_MOESM1_ESM.docx]

Additional file 1) Hypermarameter values

The model’s configuration file was constructed as follows: “attention_probs_dropout_prob": 0.1, "directionality": "bidi", "hidden_act": "gelu", "hidden_dropout_prob": 0.1, "hidden_size": 768, "initializer_range": 0.02, "intermediate_size": 3072, "max_position_embeddings": 512, "num_attention_heads": 12, "num_hidden_layers": 12, "pooler_fc_size": 768, "pooler_num_attention_heads": 12, "pooler_num_fc_layers": 3, "pooler_size_per_head": 128, "pooler_type": "first_token_transform", "type_vocab_size": 2, "vocab_size" varied by dataset. The original BERT model comprised 109,547 words, datasetA contained 10,665 words, and datasetB contained 5,893 words.

Additional file 2) Vocabulary

**Corpus (Corpora)**

A text corpus is a large and structured set of texts. A corpus may contain texts in a single language (monolingual corpus) or text data in multiple languages (multilingual corpus). “Text corpora” is the plural of “text corpus”. Nowadays it has been stored electronically and processed for various purposes of NLP. The representative corpus is Wikipedia, the largest open-collaborative online encyclopedia.

**Relation Extraction**

Relation Extraction is the task of inferring attributes and relations between entities in a given sentence. For example, from the sentence “Efficacy of acupuncture for chronic back pain”, a relation classifier aims at predicting the relation of “treatment(acupuncture)” or “disease(chronic back pain)”. As crucial step towards knowledge graph construction, Relation Extraction is a fundamental and important task for natural language processing (NLP) applications including structured search, sentiment analysis, question answering, and summarization [1].

**Data imbalance**

Data imbalance is the classification problem which occurred when one of the classes (majority class) was higher than the other classes (minority class). Imbalanced class distributions affected the training process of classifiers, leading to bias towards the majority class, resulting in high error, or even complete omission, of the minority class(es) [2].

**Over-/undersampling**

To deal with data imbalance, most prevalent approaches in the data-level includes oversampling (reducing imbalance either by creating new minority class observations) or undersampling (reducing the number of majority class observations). While undersampling can lead to insufficient learning due to lack of data, oversampling can lead to overfitting in data. Therefore, it is required to generate synthetic observations in oversampling to prevent overfitting [2, 3].

Additional file 3) Supplementary tables

Table 1. Performance of srBERT_my_ with respect to the learning steps in the first task using the original datasetA

|  | srBERT_my10K_ | srBERT_my20K_ | srBERT_my50K_ | srBERT_my100K_ | srBERT_my150K_ | srBERT_my200K_ | srBERT_my250K_ | srBERT_my300K_ | srBERT_my350K_ | srBERT_my355K_ |
| --- | --- | --- | --- | --- | --- | --- | --- | --- | --- | --- |
| AUC | 50.000 | 50.000 | 68.795 | 70.314 | 75.793 | 63.622 | 76.785 | 70.850 | 70.850 | 70.773 |
| accuracy | 89.945 | 89.945 | 93.113 | 92.562 | 93.664 | 92.562 | 94.353 | 93.526 | 93.526 | 93.388 |
| loss | 28.000 | 21.700 | 16.313 | 17.578 | 14.835 | 17.048 | 15.418 | 16.594 | 15.615 | 16.317 |
| precision | 0.000 | 0.000 | 84.848 | 72.093 | 76.471 | 95.238 | 83.333 | 86.111 | 86.111 | 83.784 |
| recall | 0.000 | 0.000 | 38.356 | 42.466 | 53.425 | 27.397 | 54.795 | 42.466 | 42.466 | 42.466 |
| F1 | 0.000 | 0.000 | 52.830 | 53.448 | 62.903 | 42.553 | 66.116 | 56.881 | 56.881 | 56.364 |

SR: Systematic Review, BERT: Bidirectional Encoder Representations from Transformers, AUC: Area under The Curve, SVC: Support Vector Classification, MultinomialNB: Multinomial Naive Bayes model

Table 2. Performance of srBERT_my_ with respect to the learning steps for the first task using the adjusted datasetA

|  | srBERT_my10K_ | srBERT_my20K_ | srBERT_my50K_ | srBERT_my100K_ | srBERT_my150K_ | srBERT_my200K_ | srBERT_my250K_ | srBERT_my300K_ | srBERT_my350K_ | srBERT_my355K_ |
| --- | --- | --- | --- | --- | --- | --- | --- | --- | --- | --- |
| AUC | 87.400 | 85.400 | 88.080 | 87.150 | 85.523 | 81.608 | 83.850 | 84.820 | 83.155 | 90.016 |
| accuracy | 87.000 | 87.000 | 88.320 | 25.278 | 87.736 | 85.600 | 86.556 | 87.500 | 86.321 | 89.380 |
| loss | 28.030 | 29.080 | 24.960 | 25.278 | 25.020 | 30.365 | 27.790 | 26.750 | 27.984 | 22.720 |
| precision | 66.279 | 67.700 | 69.380 | 68.440 | 69.900 | 66.600 | 67.690 | 69.810 | 67.567 | 68.900 |
| recall | 88.140 | 82.470 | 87.600 | 86.080 | 81.400 | 74.220 | 78.860 | 79.890 | 77.310 | 91.100 |
| F1 | 75.662 | 74.359 | 77.433 | 76.253 | 75.213 | 70.204 | 72.849 | 74.511 | 72.111 | 78.460 |

SR: Systematic Review, BERT: Bidirectional Encoder Representations from Transformers, AUC: Area under The Curve, SVC: Support Vector Classification, MultinomialNB: Multinomial Naive Bayes model

1. Huang YY, Wang WY: **Deep Residual Learning for Weakly-Supervised Relation Extraction.** [*https://arxivorg/abs/170708866*](https://arxivorg/abs/170708866) 2017.

2. Fernández A, Garcia S, Herrera F, Chawla N: **SMOTE for Learning from Imbalanced Data: Progress and Challenges, Marking the 15-year Anniversary.** *Journal of Artificial Intelligence Research* 2018, **61:**863-905.

3. Chen Y, Kempton DJ, Ahmadzadeh A, Angryk RA: **Towards Synthetic Multivariate Time Series Generation for Flare Forecasting.** 2021.
